# GFAP, CHI3L1 and GCIPL Thickness as Baseline Predictors of Early Disability Progression in MS

**DOI:** 10.3390/ijms262411774

**Published:** 2025-12-05

**Authors:** Ion Iulian Enache, Vlad Eugen Tiu, Cătălina Andreea Anghel, Cristina Tiu, Alina Popa-Cherecheanu, Mihai Bostan, Sonia Scippa, Alessia Balestrieri, Giovanni Smaldone, Andrea Soricelli

**Affiliations:** 1 Neurology Department, Emergency University Hospital Bucharest, Splaiul Independenței 169, 050098 Bucharest, Romania; ion-iulian.enache@drd.umfcd.ro (I.I.E.);; 2 Department of Clinical Neurosciences-Neurology, Carol Davila University of Medicine and Pharmacy, Bulevardul Eroii Sanitari 8, 050474 Bucharest, Romania; 3 Neurology Department, Elias University Emergency Hospital, Bulevardul Mărăști 17, 011461 Bucharest, Romania; catalina-andreea.anghel@rez.umfcd.ro; 4 Ophthalmology Department, Carol Davila University of Medicine and Pharmacy, Bulevardul Eroii Sanitari 8, 050474 Bucharest, Romania; 5 Ophthalmology Department, Emergency University Hospital Bucharest, Splaiul Independenței 169, 050098 Bucharest, Romania; 6 IRCCS SYNLAB SDN, Via G. Ferraris 144, 80146 Naples, Italy

**Keywords:** GFAP, CHI3L1, neurofilaments, biomarkers, OCT, multiple sclerosis, progression

## Abstract

Disability accumulation in multiple sclerosis often occurs independent of relapses and inflammatory activity, yet reliable predictors for early progression remain limited. Our aim was to evaluate the utility of baseline fluid and optical coherence tomography (OCT) biomarkers for predicting early disability progression in newly diagnosed relapsing–remitting MS (RRMS). We performed a monocentric observational cohort study on 72 RRMS patients that were enrolled within 6 months of diagnosis and followed for 2 years. Baseline serum and cerebrospinal fluid (CSF) samples were analyzed for neurofilament light chain (NfL), glial fibrillary acidic protein (GFAP) and chitinase-3-like protein 1 (CHI3L1). Confirmed disability progression at 1 year (1yCDP) was defined by either an increase in Expanded Disability Status Scale or a ≥20% worsening on Nine-Hole Peg Test or Timed 25-Foot Walk. Seventeen patients (23.6%) developed 1yCDP. Elevated baseline CSF GFAP (OR = 5.79, 95% CI 1.72–19.45; *p* = 0.005) and CSF CHI3L1 thickness (OR = 4.14, 95% CI 1.49–11.49; *p* = 0.006) and reduced ganglion cell-inner plexiform layer (GCIPL) thickness (OR = 0.90, 95% CI 0.84–0.97; *p* = 0.006) independently predicted 1yCDP. A multivariate model including age, CSF GFAP and GCIPL achieved AUC = 0.831, with a sensitivity of 87.5% and specificity of 61.5%. This study provides evidence that baseline patient profiling using CSF GFAP, CSF CHI3L1 and GCIPL thickness may help predict early disability progression in RRMS.

## 1. Introduction

Growing evidence indicates that the main driver of disability in patients with multiple sclerosis (pwMS) seems to be represented by progression independent of relapse activity (PIRA) [1]. The underlying mechanisms are complex and incompletely understood, and seem to include neurodegeneration, smoldering inflammation, chronic astrocytic and microglial activation, mitochondrial dysfunction and oxidative stress [2]. These processes are elusive and frequently go undetected by the regular biomarkers currently used for monitoring patients [3,4].

Neurofilament light chain (NfL) has proven its value in multiple sclerosis (MS) as a biomarker not only of disease activity through its correlation with relapse rate and presence of gadolinium-enhancing lesions, but also for prognosis and treatment response [5]. Robust evidence supports its integration into clinical practice in the near future [6].

Among the many available glial biomarkers, glial fibrillary acidic protein (GFAP) and chitinase-3-like protein 1 (CHI3L1) stand out as the most promising, particularly as markers of disability progression associated with smoldering disease [7,8,9]. GFAP has been recently described as a strong predictor of PIRA, with concentrations that increase in the presence of reactive astrogliosis and astrocytic damage [10,11]. While CHI3L1 is less explored and shows more inconsistent results, emerging data suggests that it may be a relevant CSF biomarker of PIRA [5]. This hypothesis is further supported by recent studies, showing an association between CSF CHI3L1 levels and the presence of paramagnetic rim lesions on MRI, a known indicator of smoldering disease [12]. Higher CHI3L1 levels have been detected in patients with positive cerebrospinal fluid (CSF) oligoclonal bands or elevated IgG index [13], suggesting that this biomarker may reflect ongoing glial activation and inflammatory activity within the central nervous system (CNS). However, larger cohorts are needed to determine if GFAP and CHI3L1 are reliable biomarkers of disease progression.

Optical coherence tomography has recently been added to the array of imaging modalities in MS, with both retinal nerve fiber layer (RNFL) and ganglion cell inner plexiform layer (GCIPL) thinning proving to be accurate indicators of optic nerve involvement in pwMS. Moreover, data from the Optical Coherence Tomography in Multiple Sclerosis (OCTiMS) study reported inner retinal layer loss in pwMS irrespective of a history of prior optic neuritis [14]. These results indicate that OCT measurements may detect not only neuroinflammatory processes, but also neuroaxonal degeneration. GCIPL thickness has been found to have superior reliability and reproducibility compared to RNFL thickness, proving superior correlation with both visual function and global disability scores [15].

The aim of the present study was to investigate the role of baseline fluid biomarkers (NfL, GFAP and CHI3L1) combined with OCT metrics for predicting short-term disability progression in patients with newly diagnosed relapsing–remitting multiple sclerosis (RRMS).

## 2. Results

### 2.1. Patient Characteristics

General cohort and subgroup characteristics are shown in Table 1. Of the 72 patients, 17 (23.6%) fulfilled the criteria for 1yCDP status. Patients with 1yCDP were older (median age 35 vs. 26 years, *p* = 0.003) and presented a significantly higher EDSS scores at baseline and follow-up evaluations. Except for a single case, all patients were initiated on disease-modifying therapies (DMTs) within the first month after diagnosis, most of them (77.8%) receiving moderate-efficacy DMTs. There were no significant differences between groups regarding the type of treatment (*p* = 0.399) or the number of relapses (*p* = 0.173) during the first year. Analysis of the available magnetic resonance imaging (MRI) data indicated no significant differences in MRI activity (baseline T2/FLAIR lesion load, new T2/FLAIR or gadolinium-enhancing lesions) between the two subgroups (Supplementary Table S1).

### 2.2. Biomarker Analysis

**Figure 1:**
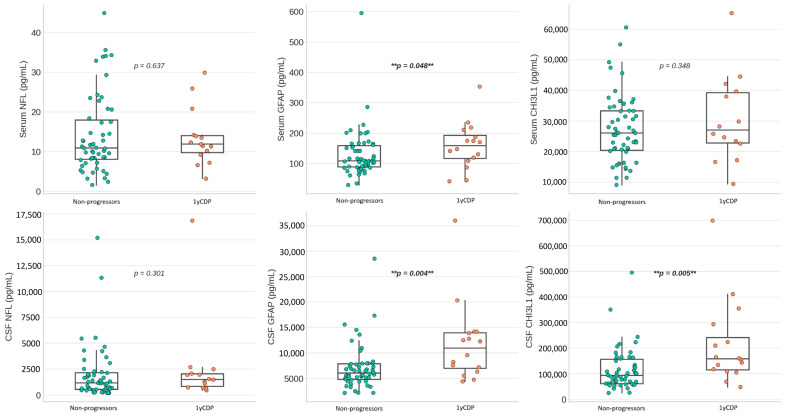
Fluid biomarker levels according to progression status (box plots with individual scatter points). ** Statistical significance for *p*-value < 0.05. CSF—cerebrospinal fluid; CHI3L1—chitinase 3-like protein 1; GFAP—glial fibrillary acidic protein; NFL—neurofilament light chain; 1yCDP—confirmed disability progression at 1 year.

Differences in biomarker profile between patients with and without 1yCDP are shown in Table 2 and Figure 1. After adjusting for covariates such as age and body mass index (BMI), significant differences were observed for CSF GFAP (*p* = 0.007), CSF CHI3L1 (*p* = 0.007), sCHI3L1 (*p* = 0.036) and ganglion cell inner plexiform layer (GCIPL) thickness (*p* = 0.003).

**Figure 2:**
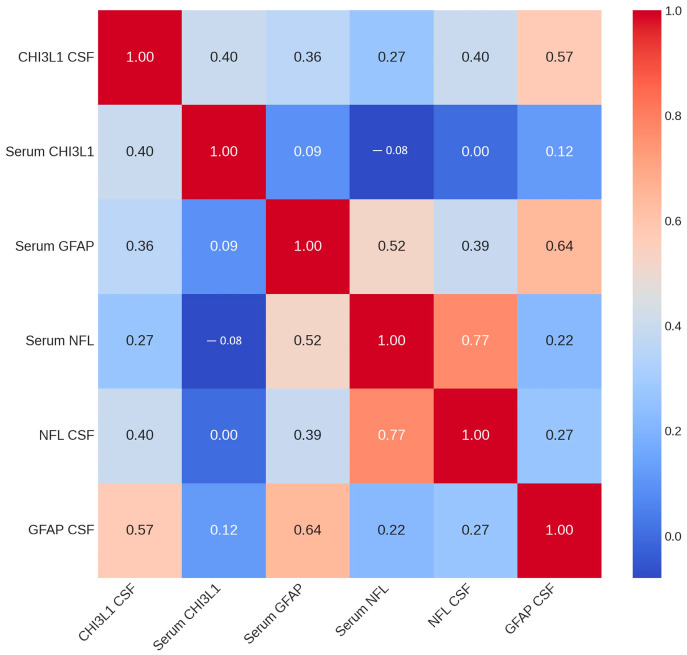
Spearman rank correlations between fluid biomarkers in the general cohort. CSF—cerebrospinal fluid; CHI3L1—chitinase 3-like protein 1; GFAP—glial fibrillary acidic protein; NFL—neurofilament light chain.

All biomarkers presented significant correlations between serum and CSF compartments in the general cohort (Figure 2). The CSF-to-serum ratio varied widely for each biomarker, with approximate mean values of 100:1 for NFL, 50:1 for GFAP and 5:1 for CHI3L1. Strong correlations were also observed between serum concentrations of GFAP and NFL (ρ = 0.524, *p* < 0.001) and between CSF levels of GFAP and CHI3L1 (ρ = 0.572, *p* < 0.001).

### 2.3. Binary Regression Analysis

Based on these primary results in baseline characteristics, we conducted univariate binary regressions using the variables which showed significant differences between subgroups to further analyze their predictive performance regarding 1yCDP status.

The selected variables used for univariate analysis are shown in Table 3. Older age (OR = 1.10, 95% CI: 1.03–1.18, *p* = 0.005) and higher baseline EDSS scores (OR = 1.75, 95% CI: 1.05–2.90, *p* = 0.03) were the clinical factors that increased the odds of 1yCDP. Biomarker data revealed that higher CSF GFAP and CHI3L1 levels at baseline presented an increased risk of progression (OR = 5.79, *p* = 0.005 and OR = 4.14, *p* = 0.006, respectively). Finally, lower GCIPL thickness was also associated with clinical disability worsening during follow-up (OR 0.90; 95% CI: 0.84–0.97; *p* = 0.006).

Multivariate binary regressions were performed to create predictive models for 1yCDP. Serum CHI3L1 and baseline EDSS did not maintain their predictive value in any multivariate regression and were therefore excluded from subsequent analysis.

**Figure 3:**
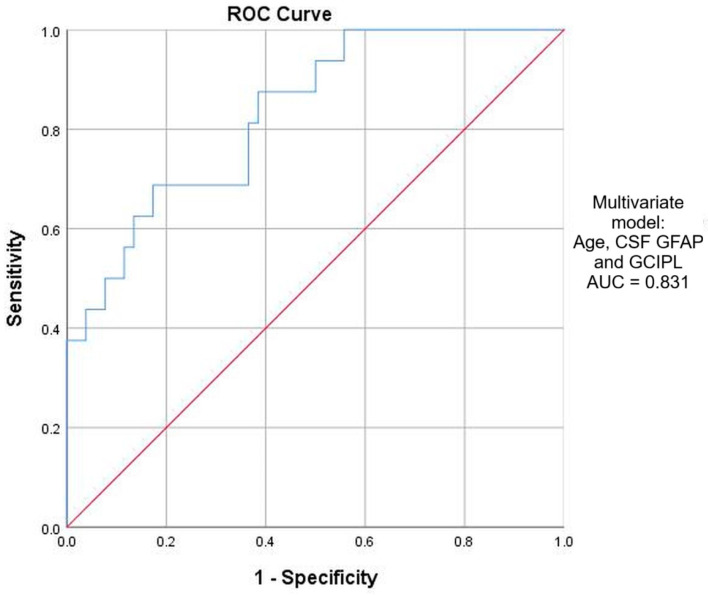
ROC curve analysis for multivariate regression model. AUC—area under curve; CSF—cerebrospinal fluid; GCIPL—ganglion cell inner plexiform layer; GFAP—glial fibrillary acidic protein.

After including age as a covariate, CSF GFAP, CSF CHI3L1 and GCIPL remained significant predictors in the multivariate regression models and demonstrated a comparable prognostic accuracy based on ROC curve analysis, with AUC values ranging from 0.799 to 0.810, indicating good model performance (see Supplementary Table S2 and Supplementary Figure S1). The best performing multivariate regression model was obtained using a three-variable model with age, CSF GFAP and GCIPL, demonstrating an AUC of 0.831 and a Nagelkerke R^2^ value of 0.385, thus indicating a moderate explanatory power (Figure 3). Based on Youden’s index, an optimal cut-off probability of 0.165 was selected for this statistical model, providing a sensitivity of 87.5% and a specificity of 61.5% for identifying patients with early disability progression. Although the collinearity diagnostics showed an elevated condition index for the three-variable model described above (CI > 30), VIF values were <1.1 for all variables in the model, indicating no harmful multicollinearity and no bias of regression coefficients (see Supplementary Tables S3 and S4). Moreover, bootstrapping analysis (1000 resamples) confirmed the stability and statistical significance of both GFAP and GCIPL.

## 3. Discussion

Neurofilament light-chain is a well-established biomarker for neuronal damage during acute disease activity in MS, but its association with smoldering activity or PIRA is still uncertain [6]. In our study, patients with 1yCDP did not show significant differences regarding baseline NfL levels, annual relapses or MRI activity compared to patients without progression, suggesting no major disparity in neuroinflammatory activity. Baseline levels of GFAP and CHI3L1 in the CSF were significantly elevated in this subgroup, raising the hypothesis that the observed disability accrual stems at least partially from mechanisms unrelated to acute inflammation, as suggested by previous work published by Meier et al. [16]. Their data reported that the differences in sNfL levels between stable patients and those with disability progression or PIRA were not statistically significant after adjusting for confounders [16]. In contrast, the same study shows that higher sGFAP levels are associated with an increased risk of disability progression and PIRA.

Emerging evidence suggests that the main driver of disability accumulation in pwMS seems to be smoldering disease activity, which may occur from very early disease stages [9]. While still incompletely understood, the underlying biological processes are considered distinct from those responsible for focal demyelination and clinical relapses. A recent international consensus paper on smoldering-associated worsening (SAW) in MS proposes GFAP and CHI3L1 as the most promising biomarkers for this novel concept [9].

Notably, in our study, CSF levels of GFAP and CHI3L1, but not NfL, demonstrated stronger correlations with 1yCDP status compared to their serum counterparts after adjustment for covariates. In univariate regression, both CSF GFAP and CHI3L1 showed a similar performance for predicting 1yCDP status. This aligns with previous reports, reflecting the compartmentalized CNS production and limited peripheral diffusion due to low blood–brain barrier permeability [17,18]. However, serum values also demonstrated a trend towards statistical significance for both biomarkers, although the small cohort size may have limited the statistical power. This raises the question of whether serum GFAP and CHI3L1 measurements could be useful for individual-level clinical decision-making, or whether their utility is rather limited to research settings for larger cohorts.

Previous work has shown that the addition of OCT metrics to clinical scores and serum biomarker levels improves the predictive power of short-term disease progression risk assessment [19]. GCIPL thickness presented more reduced values at baseline in the 1yCDP group compared to non-progressors and remained a significant predictor in multivariate regression models, which is consistent with larger longitudinal studies showing that GCIPL thickness is a strong predictor of future brain atrophy and EDSS progression [14,20].

The multivariate model including age, CSF GFAP levels and GCIPL thickness at baseline outperformed other individual predictors, achieving a sensitivity of 87.5% and a specificity of 61.5% for predicting 1yCDP status. The statistical cut-off of 0.165 based on Youden’s index was used only to provide a balanced performance of the model and was not intended to be primarily translated into clinical decision-making to classify patients at high risk for disability progression. Even though collinearity diagnostics of this model showed a high condition index for the fourth dimension, this was lower than other three-variable models. Importantly, VIF values were low and bootstrapped coefficients remained significant, indicating model stability. Although both GFAP and GCIPL are indicators of ongoing neurodegenerative processes, each captures a distinct pathophysiological mechanism. While GFAP is a biochemical indicator of glia-mediated neurodegeneration, GCIPL is a structural marker of neuronal integrity. Therefore, the addition of the two biomarkers in the same model covers complementary mechanisms occurring from early disease stages.

Our study has several limitations, mainly related to the small sample size and monocentric design. Additionally, the use of z-scores instead of raw values for GFAP and CHI3L1 would have been a more appropriate strategy in managing individual confounders such as age and BMI. Although adjustment tools have been proposed by several study groups, none are publicly available for GFAP or CHI3L1, as opposed to sNfL [21]. Despite these limitations, the main study objective was achieved, providing prospective evidence for the predictive value of GFAP, CHI3L1 and GCIPL in detecting early disease progression. Further research is needed to confirm the clinical applicability of these findings in larger cohorts and with a longer follow-up period.

## 4. Materials and Methods

We performed a monocentric, observational cohort study involving 72 patients with newly diagnosed RRMS according to the 2017 McDonald criteria [22]. Patient recruitment took place from June 2020 to June 2024 at a tertiary MS center. Inclusion criteria required an age of at least 18 years and that patients were recruited within 6 months of the moment of RRMS diagnosis. Exclusion criteria included current pregnancy or medical history that could interfere with the study protocol.

The study protocol was approved by the local Ethics Committee (registration number 12525/6), and all patients were required to sign a written informed consent form prior to inclusion.

### 4.1. Study Protocol

The study protocol included evaluations at baseline and at 1- and 2-year follow-up visits, consisting of clinical evaluation, baseline serum and CSF sampling and baseline OCT imaging. Brain MRI was performed at baseline and at 1- and 2-year follow-up in external imaging centers. All available scans were independently reviewed by two neurologists from our clinic. Clinical assessments included neurological examination and standardized functional testing using the Nine-Hole Peg Test (9HPT) and Timed 25-Foot Walk Test (T25FWT). All patients were naïve to DMTs prior to inclusion and fluid sampling. DMT was initiated within the first month after inclusion for all patients in this study. Moderate-efficacy DMTs included interferons, glatiramer acetate, teriflunomide and dimethyl fumarate, while high-efficacy DMTs included fingolimod, natalizumab, ocrelizumab and ofatumumab. All patients were followed-up with for at least 2 years after inclusion.

The clinical outcome was disability progression at 1-year follow-up (1yCDP). This was defined as either an increase in EDSS of 1.0 (for baseline EDSS ≤ 5.5) or 0.5 (for baseline EDSS > 5.5), or a ≥20% increase in 9HPT or T25FWT times. Disability progression was confirmed in year 2 of follow-up at 6 and 12 months.

### 4.2. Fluid Biomarker Analysis Protocol

Serum samples were collected after allowing blood to clot for 30–60 min, followed by centrifugation at 2000× *g* for 10 min. CSF samples were obtained after lumbar puncture and centrifuged at 400× *g* for 10 min. All samples were stored at −80 °C until further processing. Single-molecule array (SIMOA) technology (Quanterix, Billerica, MA, USA) was used for NfL and GFAP detection, while CHI3L1 levels were measured using ELLA (Biotechne, Minneapolis, MN, USA), according to the manufacturer’s instructions. NfL levels were adjusted for age and body mass index and standardized to corresponding z-scores. CHI3L1 was measured on the ELLA platform as no SIMOA assay with comparable validation is currently available for CHI3L1. To minimize platform-related variability, all samples were processed in single batches per assay, and all statistical analyses were performed within-platform, without direct comparison of concentrations across different analytical systems.

### 4.3. OCT Imaging Protocol

OCT imaging was performed in collaboration with the Ophthalmology Department of our hospital using a CIRRUS™ HD-OCT 5000 machine (Zeiss, Jena, Germany). For patients with unilateral optic neuritis (ON), we used the OCT metrics from the unaffected eye, while mean values from both eyes were used for the other patients.

### 4.4. Statistical Analysis

Statistical analysis was performed using SPSS 26.0 for Windows (SPSS Inc., Chicago, IL, USA) and Microsoft Excel 2019 (Microsoft Corp., Redmond, WA, USA). Descriptive statistics data are presented as mean ± standard deviation (SD) or as median and interquartile range (IQR). The normality of data distribution was assessed using the Kolmogorov–Smirnov test, followed by independent *t* test, Chi-square test, Fisher’s exact test and the Mann–Whitney U test for group comparisons, as appropriate. Analysis of covariance (ANCOVA) was used for group comparisons when adjusting for one or more covariates. Correlations between biomarkers were evaluated using Spearman’s rank correlation test.

Raw values of serum and CSF biomarker concentrations were used for group comparison and correlations. Prior to regression analyses, raw biomarker values were transformed using the natural logarithm to address skewness and the wide range of concentrations, as well as reduce the influence of outliers. Univariate binary logistic regression was performed to evaluate the predictive value of each variable. Multivariate binary regressions were used for prediction models regarding 1yCDP status. To assess the stability of the regression models, non-parametric bootstrapping was performed. The bootstrapped coefficients were consistent with the original results, showing minimal bias and remaining statistically significant. Multicollinearity was evaluated using Variance Inflation Factor (VIF) and collinearity diagnostics (condition index and variance proportions). A *p*-value ≤ 0.05 was considered statistically significant. Subjects with missing data were excluded from the analyses.

## 5. Conclusions

Confirmed disability progression at 1 year occurred for 23.6% of patients with newly diagnosed RRMS in our study. Older age and higher EDSS score at the time of diagnosis, as well as higher baseline levels of GFAP and CHI3L1, but not NfL, were independent predictors of early disability progression. Moreover, combining CSF GFAP with GCIPL thickness from retinal imaging enhanced predictive accuracy over other tested models, supporting the potential value of this multimodal approach for patient profiling.

Our data suggest that disability progression occurs largely independent of neuroinflammatory activity of the disease, as no significant differences were observed between subgroups regarding clinical, imaging and serum biomarkers related to inflammatory activity. These real-world findings reinforce the growing evidence that disability progression is common very early after diagnosis for a significant percentage of RRMS patients, and this subgroup may be identified at baseline using accessible biomarkers, with potential implications for early management strategies.

## Figures and Tables

**Table 1:** Cohort characteristics according to progressor status.

Variable	Total RRMS Patients (N = 72)	Non-1yCDP (N = 55)	1yCDP (N = 17)	*p*-Value
Age, years (median, IQR)	29 (23–35)	26 (21–33)	35 (29–43)	0.003 *
Gender, % (female)	75%	72.7%	82.3%	0.533
Smoking status, % (active)	29.2%	28.6%	31.3%	0.901
Duration from first symptom onset, years (median, minimum and maximum)	1 (0, 12)	1 (0, 12)	0 (0, 7)	0.225
Type of treatment during first year, N				
No treatment	1 (1.4%)	1 (1.8%)	0 (0%)	
Moderate efficacy	56 (77.8%)	41 (74.6%)	15 (88.2%)	0.399
High efficacy	15 (20.8%)	13 (23.6%)	2 (11.8%)	
Number of relapses in the first year, N				
No relapse	49 (68%)	40 (72.7%)	9 (52.9%)	
1 relapse	21 (29.2%)	13 (23.6%)	8 (47.1%)	0.173
2 relapses	2 (2.8%)	2 (3.7%)	0 (0%)	
EDSS at baseline (median, IQR)	1.5 (1.5–2.0)	1.5 (1.5–2.0)	2.0 (1.5–3.5)	0.049 *
EDSS at 1-year follow-up (median, IQR)	1.5 (1.0–2.0)	1.5 (1.0–2.0)	3.0 (1.5–4.5)	<0.001 *
Mean EDSS change (mean, SD)	−0.04 ± 0.88	−0.25 ± 0.65	0.61 ± 1.2	0.001 *
EDSS at 2-year follow-up (median, IQR)	1.5 (1.5–2.5)	1.5 (1.5–2.0)	3.0 (1.5–5.0)	<0.001 *
T25FWT at baseline, seconds (median, IQR)	6.17 (5.37–7.08)	6.18 (5.67–7.09)	5.75 (4.49–7.08)	0.208
T25FWT at 1-year follow-up, seconds (median, IQR)	5.5 (5.15–5.85)	5.4 (5.0–5.7)	5.8 (5.48–6.98)	0.002 *
Mean change in T25FWT, seconds (mean, SD)	−0.5 ± 2.29	−1.15 ± 1.4	1.61 ± 3.23	<0.001 *
9HPT dominant hand at baseline, seconds (median, IQR)	19.7 (17.6–22.1)	19.73 (17.8–22.1)	18.5 (17.6–22.3)	0.937
9HPT dominant hand at 1-year follow-up, seconds (median, IQR)	17.8 (16.8–18.9)	17.6 (16.6–18.4)	18.8 (17.1–21.8)	0.027 *
9HPT non-dominant hand at baseline, seconds (median, IQR)	21.25 (19.15–23.9)	21.3 (19.7–23.8)	20.4 (18.7–25.1)	0.776
9HPT non-dominant hand at 1-year follow-up, seconds (median, IQR)	19.4 (18.0–23.4)	19.1 (18.0–21.7)	20.5 (18.9–25.4)	0.071
Mean change in 9HPT dominant hand, seconds (mean, SD)	−1.61 ± 3.4	−2.27 ± 2.74	0.5 ± 4.4	0.003 *
Mean change in 9HPT non-dominant hand, seconds (mean, SD)	−1.68 ± 4.58	−2.24 ± 4.56	0.16 ± 4.21	0.058

* Statistical significance for *p*-values < 0.05 (in bold); EDSS—Expanded Disability Status Scale; IQR—interquartile range; N—number; RRMS—relapsing–remitting multiple sclerosis; SD—standard deviation; T25FWT—Timed 25-Foot Walk Test; 1yCDP—1-year confirmed disability progression; 9HPT—Nine-Hole Peg Test.

**Table 2:** Biomarker profiling according to disability progression status.

Biomarker	Non-1yCDP	1yCDP	Unadjusted *p*-Value	Adjusted *p*-Value (ANCOVA) *
sNFL, pg/mL (median, IQR)	10.9 (7.9–18.4)	11.9 (9.2–14.2)	0.637	0.758
sNFL z-score (median, IQR)	2.1 (0.92–2.65)	1.77 (0.81–2.46)	0.678	-
CSF NFL, pg/mL (median, IQR)	1151 (507–2156)	1498 (718–2156)	0.301	0.657
sGFAP, pg/mL (median, IQR)	109.24 (88.0–161.2)	159.2 (111.8–205.1)	**0.048 ^†^**	0.496
CSF GFAP, pg/mL (median, IQR)	6085.3 (4811–7930)	10,932 (6578–14,105)	**0.004 ^†^**	**0.007 ^†^**
sCHI3L1, pg/mL (median, IQR)	26,064 (20,235–33,378)	27,030 (21,280–40,312)	0.348	**0.036 ^†^**
CSF CHI3L1, pg/mL (median, IQR)	93,019 (61,483–157,635)	157,682 (110,797–275,447)	**0.005 ^†^**	**0.007 ^†^**
RNFL, µm (median, IQR)	95 (87.5–102)	92 (82–97.5)	0.103	0.103
GCIPL, µm (median, IQR)	82 (75.5–86)	76 (67.8–79.5)	**0.003 ^†^**	**0.009 ^†^**

* Serum biomarkers were adjusted for age and BMI; all other biomarkers were adjusted only for age. ^†^ *p*-values < 0.05 (bold) were considered statistically significant. ANCOVA—analysis of covariance; CSF—cerebrospinal fluid; CHI3L1—chitinase 3-like protein 1; GCIPL—ganglion cell inner plexiform layer; GFAP—glial fibrillary acidic protein; NfL—neurofilament light chain; RNFL—retinal nerve fiber layer; sCHI3L1—serum CHI3L1; sGFAP—serum GFAP; sNfL—serum NfL; 1yCDP—confirmed disability progression at 1 year.

**Table 3:** Binary logistic regression—univariate and multivariate models for predicting 1yCDP.

	Univariate		Multivariate (Best Model)	
Variable	OR (95% CI)	*p*-Value	OR (95% CI)	*p*-Value
Age	1.1 (1.03–1.18)	**0.005** *	1.08 (1.0–1.17)	**0.037** *
Baseline EDSS	1.75 (1.05–2.9)	**0.03** *	-	-
Serum CHI3L1 (ln-transformed)	1.68 (0.40–7.02)	0.477	-	-
CSF GFAP (ln-transformed)	5.79 (1.72–19.45)	**0.005** *	4.48 (1.26–15.9)	**0.021** *
CSF CHI3L1 (ln-transformed)	4.14 (1.49–11.49)	**0.006** *	-	-
GCIPL	0.9 (0.84–0.97)	**0.006** *	0.91 (0.83–1.0)	**0.038** *

* Statistical significance at *p*-value threshold <0.05 (bold). CHI3L1—chitinase 3-like protein 1; CI—confidence interval; CSF—cerebrospinal fluid; EDSS—Expanded Disability Status Scale; GCIPL—ganglion cell-inner plexiform layer; GFAP—glial fibrillary acidic protein; OR—odds ratio.

## Data Availability

The data analyzed in this study are not publicly available due to patient confidentiality but may be provided by the corresponding author upon reasonable request.

## Supplementary Materials

The following supporting information can be downloaded at: https://www.mdpi.com/article/10.3390/ijms262411774/s1.

## Abbreviations

The following abbreviations are used in this manuscript:

Abbreviation	Definition
1yCDP	One-year confirmed disability progression
AUC	Area under the curve
ANCOVA	Analysis of covariance
BMI	Body mass index
CDP	Confirmed disability progression
CHI3L1	Chitinase-3-like protein 1
CI	Confidence interval
CNS	Central nervous system
CSF	Cerebrospinal fluid
DMT	Disease-modifying therapy
EDSS	Expanded Disability Status Scale
GCIPL	Ganglion cell inner plexiform layer
GFAP	Glial fibrillary acidic protein
IQR	Interquartile range
MRI	Magnetic resonance imaging
MS	Multiple sclerosis
NfL	Neurofilament light chain
OCT	Optical coherence tomography
ON	Optic neuritis
OR	Odds ratio
PIRA	Progression independent of relapse activity
pwMS	Patient with multiple sclerosis
RNFL	Retinal nerve fiber layer
ROC	Receiver operating characteristic
SD	Standard deviation
SIMOA	Single-molecule array
sCHI3L1	Serum chitinase-3-like protein 1
sGFAP	Serum glial fibrillary acidic protein
sNfL	Serum neurofilament light chain
SPSS	Statistical Package for the Social Sciences
T25FWT	Timed 25-Foot Walk Test
9HPT	Nine-Hole Peg Test
