# Supplementary material for: GFAP, CHI3L1 and GCIPL Thickness as Baseline Predictors of Early Disability Progression in MS
Source: Int J Mol Sci. 2025 Dec 5;26(24):11774. doi: 10.3390/ijms262411774 (PMC12733348; doi:10.3390/ijms262411774)
Supplement: Supplementary file 1 [file ijms-26-11774-s001.zip › ijms-3987208-supplementary.pdf]

Supplementary Table S1. Cohort MRI characteristics according to progressor status

**Variable**	**Available scans,** N (%)	**Non-1yCDP,** N (%)	**1yCDP,** N (%)	***p* value**	**RR** (95% CI)
≥ 9 T2/FLAIR lesions at baseline	53 (73.61%)	32/42 (76.2%)	11/11 (100%)	0.098	0.76 (0.64-0.90)
≥ 1 contrast-enhancing lesion at baseline	41 (56.94%)	15/31 (48.4%)	3/10 (30%)	0.467	1.61 (0.58-4.44)
≥ 3 new T2/FLAIR lesions at 1 year follow-up	48 (66.66%)	8/37 (21.6%)	2/11 (18.2%)	>0.99	1.18 (0.29-4.80)
≥ 1 contrast-enhancing lesion at 1 year follow-up	36 (50%)	4/27 (14.8%)	1/9 (11.1%)	>0.99	1.33 (0.17-10.43)

Note: p-values < 0.05 were considered statistically significant; CI - confidence interval; FLAIR - fluid-attenuated inversion recovery; N - number; RR - relative risk; 1yCDP - 1 year confirmed disability progression

Supplementary Table S2. Multivariate regression models for predicting 1yCDP status

	**OR (95% CI)**	**p-value**	**AUC**	**Nagelkerke R^2**
**Model 1**			0.810	0.306
• Age	1.08 (1.01 – 1.16)	0.026		
• CSF GFAP (ln-transformed)	5.24 (1.47 – 18.6)	0.01		
**Model 2**			0.804	0.291
• Age	1.08 (1.01 – 1.16)	0.023		
• CSF CHI3L1 (ln-transformed)	3.79 (1.30 – 11.05)	0.014		
**Model 3**			0.799	0.297
• Age	1.1 (1.02 – 1.81)	0.011		
• GCIPL	0.90 (0.83 – 0.98)	0.015		
**Model 4**			0.831	0.385
• Age	1.08 (1.0 – 1.17)	0.037		
• CSF GFAP (ln-transformed)	4.48 (1.26 – 15.9)	0.021		
• GCIPL	0.91 (0.83 – 1.0)	0.038		

Note: p-values < 0.05 were considered statistically significant; OR – odds ratio; AUC – area under curve; CSF – cerebrospinal fluid; GFAP – glial fibrillary acidic protein; CHI3L1 – chitinase 3-like 1; GCIPL – ganglion cell – inner plexiform layer

Supplementary Figure S1. ROC curve analysis for the 2-variable regression models

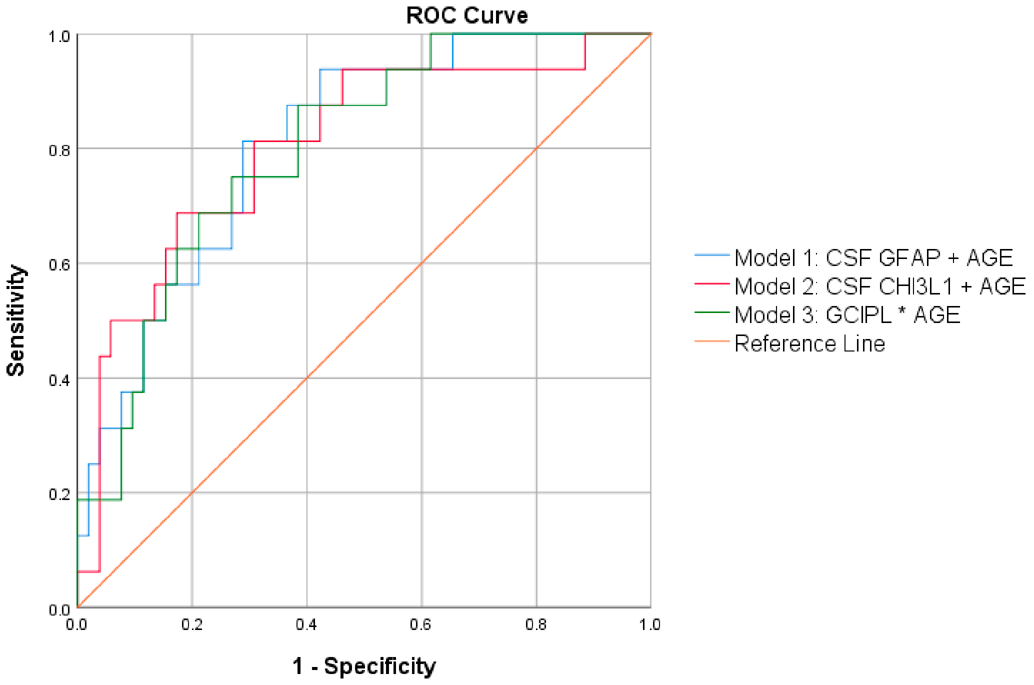

Note: ROC - receiver operating characteristic; CSF – cerebrospinal fluid; GFAP – glial fibrillary acidic protein; CHI3L1 – chitinase 3-like 1; GCIPL – ganglion cell – inner plexiform layer.

Supplementary Table S3. Bootstrap estimates and collinearity statistics for logistic regression coefficients in the 3-variable prediction model (*Model 4*)

		Bootstrap					Collinearity Statistics	
Variable	B	Bootstrap Bias	Standard error	Bootstrap p-value	95% CI (lower)	95% CI (upper)	Tolerance	VIF
Age	0.082	0.018	0.140	0.019	0.013	0.195	0.952	1.051
CSF GFAP	1.499	0.598	11.44	0.008	0.373	3.681	0.919	1.088
GCIPL	-0.092	-0.025	0.447	0.013	-0.222	-0.012	0.920	1.087

Note: p-values < 0.05 were considered statistically significant; CSF – cerebrospinal fluid; GFAP – glial fibrillary acidic protein; GCIPL – ganglion cell – inner plexiform layer; CI – confidence interval; VIF - variance inflation factor.

Supplementary Table S4. Multicollinearity analysis for the 3-variable prediction model (*Model 4)*

				Variance Proportions			
Model	Dimension	Eigenvalue	Condition Index	(Constant)	Age	CSF GFAP	GCIPL
4	1	3.926	1.000	0.00	0.00	0.00	0.00
	2	0.064	7.826	0.00	0.89	0.00	0.02
	3	0.009	21.346	0.01	0.10	0.16	0.64
	4	0.001	53.241	0.98	0.00	0.84	0.34

Note: a. Additional 3-variable models were evaluated – (1) age, CSF CHI3L1 and GCIPL, as well as (2) age, CSF CHI3L1 and CSF GFAP. Both these models exhibited significantly higher multicollinearity based on condition indexes (63.35 in Dimension 4, respectively 44.84 in Dimension 3 and 54.95 in Dimension 4) and variance proportions and therefore were not included in further analysis.

b. Although the collinearity diagnostics showed an elevated condition index, this is expected given the correlation between GFAP and GCIPL, which reflect related biological processes in MS (glial activation and neuroaxonal degeneration). Importantly, VIF values were <1.1 for all variables in the model, indicating absence of harmful multicollinearity and no bias of regression coefficients (see **Table S3**). Moreover, bootstrapping analysis (1000 resamples) confirmed the stability and statistical significance of both GFAP and GCIPL. For these reasons, both biomarkers were kept in the multivariate model, as they represent complementary and clinically relevant aspects of MS pathology.
